# Knowledge-sharing hostility, knowledge manipulation, and new product development performance

**DOI:** 10.3389/fpsyg.2022.793712

**Published:** 2022-09-12

**Authors:** Ruilin Cai, Yingshuang Ma

**Affiliations:** ^1^School of Business, Changshu Institute of Technology, Suzhou, China; ^2^School of Business, Huanggang Normal University, Huanggang, China

**Keywords:** knowledge sharing, knowledge-sharing hostility, knowledge manipulation, coordination flexibility, new product development

## Abstract

New product development is an important driver of sustainable enterprise development. It is necessary to promote the knowledge sharing of heterogeneous individuals such as design, technology, market, and sociologists. This paper discusses the influence of negative individual knowledge management from the perspective of knowledge-sharing hostility and knowledge manipulation on the performance of new product development. To examine our hypotheses, we conducted a questionnaire survey of 438 employees in China. The results show that although knowledge manipulation contributes to individual innovation performance, it has an inverted U-shaped curve relationship with the team's product development performance. The hostility of knowledge sharing induces knowledge manipulation, which indirectly influences the performance of new product development. The coordination flexibility of R&D teams positively moderates the impact of knowledge manipulation on new product development. Implications and future research directions are discussed.

## Introduction

New product development is a determining factor for enterprises to maintain a competitive advantage and sustainable development. The process of the design and development of a new product is complex and full of risks (Goswami et al., 2021). The efficiency of new product development mainly depends on the environmental mechanisms and process mechanisms (Najafi-Tavani et al., 2018). Specifically, the environmental mechanism includes enterprise designers, technicians, sociologists, market experts, and other design and research members, and it includes communication mechanisms. The process contains the adoption, interpretation, and expression of various types of knowledge (Dell'Era and Verganti, 2009). Thus, the importance of knowledge sharing is determined by the complexity of new product development tasks, the heterogeneity of subject knowledge, and the unbalanced distribution of knowledge.

Knowledge is an important support for the survival and development of employees, and knowledge sharing is increasingly acknowledged as a core competence for individual creativity and innovation performance (Song et al., 2022; Wang et al., 2022). Moreover, knowledge sharing is not only the process of knowledge transfer but also the interaction between sender and receiver of knowledge (Yao et al., 2021). Therefore, effective knowledge sharing provides individuals, teams, and organizations with opportunities to improve work performance and create new ideas Gao and Bernard, 2018; Zhao et al., 2019). However, owing to the complexity of knowledge sharing, many individual, organizational, and contextual factors may influence it (Ahmad and Karim, 2019). Once knowledge has been shared, it becomes a public product that other colleagues can obtain and use. Given its growing importance, scholars have examined various knowledge sharing of knowledge sharing, including organizational atmosphere, organizational incentive plans, knowledge attributes, sharing methods, and the support of information technology (Al-Alawi et al., 2007; Jain et al., 2015; Yamauchi, 2015).

Knowledge hiding, which may lead to the widespread hostility of knowledge sharing between individuals, refers to the intentional concealment or refusal of employees to provide other people's knowledge requests (Zhao et al., 2019). Connelly et al. (2012) found that knowledge hiding negatively affects the employees' creativity and individual status. In other words, hiding behavior negatively affects innovation performance and hinders individuals from gaining trust and personal influence (Zhao et al., 2019). Taking into account the negative effects of knowledge hiding, Research and Development (R&D) professionals usually do not choose complete knowledge hiding or sharing; they will intelligently select individual knowledge-sharing strategies, namely, knowledge manipulation. Prior research has primarily focused on the antecedents of knowledge-sharing hostility and knowledge manipulation from the perspectives of goal orientation, team culture, leadership style, and knowledge ownership, but the research on innovation performance is underexplored. To discuss the relationship among knowledge-sharing hostility, knowledge manipulation, and the performance of new product development, we explore the impact of knowledge manipulation on the development performance of new products under the hostility of knowledge sharing. This study contributes to the literature in two ways. First, it contributes to the theory of knowledge sharing by making the mechanism from knowledge manipulation to innovation performance clear. Second, our study can provide references for new product development. Specifically, we found that knowledge-sharing hostility and knowledge manipulation are negatively related to individual knowledge management.

## Theory and hypothesis

### Knowledge-sharing hostility and knowledge manipulation

Knowledge-sharing hostility refers to knowledge hoarding, knowledge exclusion, and a negative sharing failure attitude. From the perspective of knowledge ownership, knowledge (especially tacit knowledge) is rooted in individuals and is an essential factor in individual competition. Therefore, individuals have knowledge-sharing hostility, hindering individual knowledge-sharing behavior. However, people are included in different organizations, and organizations require their members to share the necessary knowledge, which will lead to the conflict of knowledge ownership perception between individuals (Rechberg and Syed, 2013). Therefore, although individuals are hostile to knowledge sharing, knowledge sharing is still needed.

The concept of knowledge manipulation originated from Bettis-Outland (1999), who found that the accuracy of information is influenced by some human factors. To be exact, people will operate knowledge based on the performance objectives. Knowledge-based people selectively share knowledge based on their interests and neither complete knowledge sharing nor pure knowledge hiding (Rhee and Choi, 2017). Therefore, we define knowledge manipulation as a selective and even misleading sharing behavior that maximizes personal interests.

In the practice of new product development, R&D personnel want to obtain their interests or reflect their value through knowledge sharing. However, they may manipulate or hide knowledge when they lack the necessary benefits. In addition, because R&D personnel are in the R&D team, their organizational identity requires members to share the necessary knowledge. This may lead to the concealment of important information or salient knowledge, although R&D personnel obey the concept of organizational knowledge sharing. Therefore, employees with knowledge-sharing hostility may be more likely to induce knowledge manipulation for their interests.

*H*_1_*: Knowledge-sharing hostility positively affects knowledge manipulation*.

Knowledge hoarding refers to the employees accumulating knowledge without sharing knowledge, positively impacting knowledge operations (Lee et al., 2011). People can exchange knowledge as personal knowledge or experience. In addition, resource-based theory holds that unique, valuable, and challenging to replicate resources are the premise of maintaining competitive advantage. Therefore, to obtain the expected return, people may cover, exaggerate, or treat value differently in knowledge sharing (Ford and Staples, 2010). Moreover, product development is not a single activity requiring multiparty cooperation, such as technology, design, marketing, production, sociology, and psychology. In a competitive relationship, to maintain competitive advantage, enterprise employees continue to hoard the quantity and quality of knowledge, resulting in increasing space for them to manipulate knowledge. Therefore, individuals who collect more knowledge are more likely to induce opportunism through knowledge sharing. Based on this, the following assumptions are put forward:

*H*_1*a*_*: Knowledge hoarding positively affects knowledge manipulation*.

Knowledge exclusion is based on the concept of knowledge recipient. Specifically, the behavior of knowledge recipients refusing to share knowledge with others is considered a “non-invention syndrome” (Katz and Allen, 1982). After that, Husted et al. (2012) further explained the reasons for knowledge exclusion: first, the recipient may have an attitude of resisting the knowledge provided by others; second, they will question the authenticity or value of learning from others; third, they are not familiar with the knowledge taught by others. In new product development, knowledge exclusion will affect employees' knowledge manipulation. Considering that knowledge manipulation aims to maximize personal interests (Peng, 2013), when employees realize knowledge exclusion, they must seek higher knowledge manipulation skills to maximize the private interests of knowledge manipulation.

*H*_1*b*_*: Knowledge exclusion positively affects knowledge manipulation*.

New product development is full of challenges and risks, and there exists the possibility of failure to adopt and apply knowledge. Knowledge providers' awareness of risk will significantly weaken their willingness to share knowledge. Tseng (2010) succinctly summed up the concerns about potential accountability for sharing failure as a “negative attitude toward sharing failure.” To gain individual interests and reduce the risk of failure, R&D employees may conduct knowledge manipulation more attentively. In other words, the negative sharing failure attitude will promote the behavior of knowledge manipulation more attentively. Based on this, the following hypothesis is proposed:

*H*_1*c*_*: Negative sharing failure attitude positively affects knowledge manipulation*.

### Knowledge manipulation and new product development performance

Moderate knowledge manipulation has a positive impact on improving individual innovation performance. Although the primary purpose of knowledge manipulation is to maximize the interests of knowledge operators, it helps to protect the interests of actors at the level of individual innovation performance to form a manipulator-centered knowledge network structure and continuously increase all the aspects of benefits for manipulators. Moreover, the study found that knowledge manipulation can expand the status or influence of knowledge manipulators (Wang and Noe, 2010). From a dialectical point of view, although knowledge operation exaggerates or misleads the value of knowledge, the manipulator improves the attention of knowledge and stimulates creativity through the strategic manipulation of important packaging information. At the same time, it can further enhance the influence and enthusiasm of the manipulator. Therefore, it can be seen that although the operators cover up their real purpose, they do not exclude knowledge sharing. However, operators still strategically choose knowledge sharing, which objectively makes individuals improve their innovation performance and increases their traditional role in new product development technology, product meaning, and markets. In other words, appropriate knowledge manipulations can enhance recent product development performance in design and development (Donate and Pablo, 2015; Good et al., 2022).

This result agrees with the positive impact of knowledge manipulation, but its negative impact cannot be ignored. The common manifestation of knowledge manipulation is that individuals exaggerate expectations, ignore or dilute potential defects, and improve the expected value of knowledge. Excessive knowledge manipulation may lead to the maximization of individual interests and the reduction in collective goods (Steinel et al., 2010). In new product development, demand management is regarded as a critical factor, which includes direct awareness of demand, predictable demand, and unpredictable demand, making this process full of challenges and risks (Aydin et al., 2014). Suppose knowledge manipulators often fail to achieve the expected value. In that case, it may be attributed to the intrinsic motivation of the manipulators and even doubt the knowledge-sharing motivation of other members, thus undermining the trust between each other and the collaborative atmosphere of innovation. Moreover, it may lead R&D personnel to ignore the potential risks of new product development and ultimately harm recent product development performance. Based on this, the following assumptions are put forward:

*H*_2_*: There is an inverted U-shaped curve relationship between knowledge manipulation and new product development performance*.

### The mediating role of knowledge manipulation

Effective knowledge sharing can realize the effect of collaborative innovation. Empirical studies have found that knowledge sharing can enhance the innovation capabilities of employees, teams, and the entire organization (Darroch, 2005; Sun and Jin, 2012; Gilson et al., 2013). For example, knowledge sharing plays an important role in the relationship between technical knowledge and product function design and product semantic innovation (Zhao et al., 2019). However, knowledge-sharing hostility significantly negatively affects individual knowledge sharing (Husted et al., 2012). When an employee has knowledge-sharing hostility, it will weaken the ability to innovate through negative influences on knowledge sharing. Therefore, once employees have knowledge-sharing hostility, it will inevitably induce knowledge hoarding, knowledge exclusion, and negative sharing failure attitudes, negatively affecting new product development performance. Based on this, the following hypothesis is proposed:

*H*_3_*: Knowledge-sharing hostility negatively affects new product development performance*.

Knowledge-sharing hostility includes three aspects: knowledge hoarding, knowledge exclusion, and negative attitudes toward sharing failure. The study found that knowledge-sharing hostility is an antecedent variable of knowledge manipulation and impacts new product development performance. Researchers examined the mediating effects of knowledge behaviors (e.g., knowledge manipulation) of goal orientation on individual innovation performance through sample data of 214 employees in 37 teams (Rhee and Choi, 2017). New product development requires the flexible formation of different types of employees, who work together to complete tasks. However, there exists a mutual competition and cooperation relationship between them because the relevant individuals have differences in profession and determine the inevitable existence of knowledge-sharing hostility. Furthermore, knowledge-sharing hostility induces knowledge manipulation and then affects new product development performance through knowledge manipulation. Based on this, the following hypothesis is proposed:

*H*_4_*: Knowledge manipulation plays a mediating role between knowledge-sharing hostility and new product development performance*.

### The moderating role of coordination flexibility

In a rapidly changing environment where companies need to adapt to environmental changes, the process of new product development is full of uncertainty. Moreover, overall knowledge manipulation will lead to the destruction of innovation culture and weaken new product development performance if employees do not perceive the knowledge of the operator to provide the expected value of knowledge (Tenbrunsel, 1998). To make full use of existing knowledge resources, enterprises should make necessary interventions to influence the negative influence through strategic flexibility, which is divided into resource flexibility and coordination flexibility (Nadkarni and Narayanan, 2007). Resource flexibility refers to the flexibility of existing resources, the availability of idle resources, and the creation and accumulation of potential resources. Knowledge manipulation is essentially a knowledge-sharing strategy, and the relationship between it and the performance of new product development may be adjusted by coordination and flexibility.

The appeal of coordination and flexibility can improve the utilization efficiency of existing innovation resources and explore ways of using existing innovation resources. When the knowledge-sharing strategy conforms to the goal of coordination and flexibility, knowledge manipulation is regarded at a moderate level. In this way, coordination flexibility amplifies the positive relationship between knowledge manipulation and new product development performance. However, when knowledge manipulation negatively affects innovation performance, it leads to incompatibility with coordination flexibility and the dilemma of the knowledge-sharing dilemma (Kimmerle and Wodzicki, 2011). Specifically, the incompatibility of coordination and flexibility will induce individuals to protect professional knowledge and promote the behavior of knowledge manipulation and knowledge-sharing hostility, ultimately negatively impacting innovation performance. In addition, excessive coordination flexibility may excessively influence a harmonious innovation soft environment and high bond strength and lead to the improvement in organizational innovation performance (Han and Zhang, 2021). In summary, we put forward the following hypothesis:

*H*_5_*: Coordination flexibility moderates the relationship between knowledge manipulation and new product development performance*.

### The moderated mediation effect

We assume that knowledge-sharing hostility has an indirect impact on new product development performance through knowledge manipulation and that coordination flexibility moderates the relationship between knowledge manipulation and new product development performance. Given the above, this study further points out that coordination flexibility moderates the mediating role of knowledge manipulation in the development of new products. Previous research found a significant positive influence of knowledge manipulation (Erdem and Mustafa, 2009; Wang and Noe, 2010), which is consistent with the goal of coordination flexibility. Furthermore, coordination flexibility enhances the indirect effect of knowledge manipulation on knowledge-sharing hostility. Therefore, we propose the following:

*H*_6_*: Coordination flexibility moderates the relationship between knowledge-sharing hostility and new product development performance via knowledge manipulation*.

**Figure 1:**
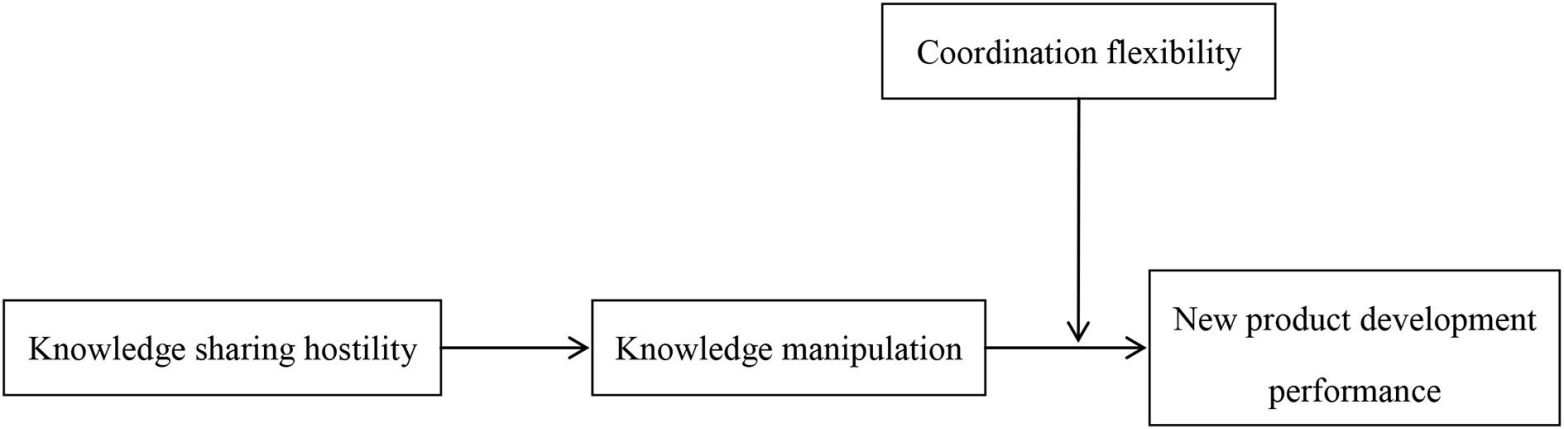
Research framework.

The research framework is shown in Figure 1.

## Method

### Sample and procedures

To examine the hypotheses, we collected data from designers and researchers enrolled in different industries in China. We sent the survey packages to employees *via* e-mail or postal mail. Specifically, we solicited voluntary participation from 1,000 employees, who occupied designers and researchers positions in their organizations, representing a broad range of industries, including furniture design, textile and garment, electronic equipment, culture and education, and food and beverage. Of the 1,000 employees, 457 agreed to participate in the survey. After removing sixteen members without key information, we obtained a final analysis sample of 438 employees.

The employee sample included 49.3% women. They are from different industries, including furniture design (22.4%), textiles and garments (20.1%), electronic equipment (22.1%), culture and education (18.0%), and food and beverages (17.4%). The age levels of the employees were under the age of 30 (34.5%), 31–40 (34.5%), and over 41 years old (31%). The educational levels of the employees were high school (9.6%), undergraduate degree (46.2%), and graduate degree (44.3%).

### Measurements

To ensure the reliability and validity of the measurements, scales that have been validated are selected and are revised slightly in combination with new product development. All items were rated on a five-point Likert scale ranging from “strongly disagree” to “strongly agree.”

#### Knowledge-sharing hostility

We adopted nine items from Husted et al. (2012) to assess knowledge sharing. One sample item was “I often do not trust knowledge sources outside my department.” Cronbach's alpha coefficient was 0.713.

#### Knowledge manipulation

We assessed knowledge manipulation using the scale validated by Rhee and Choi (2017). This scale comprised of four items, and the sample item was “I equivocated with the core information while explaining my knowledge.” Cronbach's alpha coefficient was 0.806.

#### Coordination of flexibility

We constructed a four-item measure of coordination of flexibility based on the previous studies (Tenbrunsel, 1998; Nadkarni and Narayanan, 2007). Specifically, they rated four items following this instruction: “the company allows to break the formal working procedures to maintain the flexibility and dynamics of new product development; the design and development work patterns vary from person to person, tailored to the times; there are very smooth communication channels and mechanisms in new product development; the company can actively and proactively respond to external competition.” Cronbach's alpha coefficient was 0.890.

#### New product development performance

We assessed new product development performance using the scales validated by Genç and Benedetto (2015). It comprised of five items, and the sample item was “the new product design and development cycle is shorter than the competition.” Cronbach's alpha coefficient was 0.710.

## Results

### Data analysis

Prior to hypothesis testing, we conducted the Harman single-factor method on 22 measurement items of four latent variables. We performed an exploratory factor analysis on Statistical Package for the Social Sciences (SPSS) and constrained the number of factors extracted to one factor with no rotation. The results indicated that common method bias was not a concern in this study since less than 50% of the variance (20.92%) was explained by the single factor. To reduce the possible multicollinearity problem of the regression equation, four latent variables, such as knowledge-sharing hostility, are centralized.

### Reliability and validity analysis

The exploratory factor analysis of knowledge-sharing hostility was carried out by principal component analysis. The results showed that the Kaiser–Meyer–Olkin (KMO) coefficient was 0.724, and the Bartlett's spherical test results passed the test at the 0.001 level, indicating that the exploratory factor analysis (EFA) method was appropriate. The factor extraction values of the 9 items are between 0.668 and 0.829, and the three main factors are extracted according to the eigenvalues greater than 1. The first principal factor eigenvalue is 2.821, corresponding to the three items of knowledge accumulation. The second principal factor eigenvalue is 2.063, corresponding to the three items of knowledge exclusion; the third principal factor eigenvalue is 1.912, corresponding to the negative shared failure attitude. The cumulative variance interpretation rate of the three principal factors is 75.507%, indicating that the overall information on knowledge-sharing hostility is well reflected. The details are shown in Table 1.

**Table 1:** Exploratory factor analysis of knowledge-sharing hostility.

**Indicators**	**Factor 1**	**Factor 2**	**Factor 3**
A1	**0.908**	0.038	0.050
A2	**0.912**	0.095	0.044
A3	**0.899**	0.058	0.056
B1	0.039	**0.834**	−0.001
B2	0.117	**0.874**	0.054
B3	0.026	**0.847**	−0.011
C1	−0.059	−0.032	**0.827**
C2	0.128	0.005	**0.860**
C3	0.078	0.064	**0.811**

*The bold values in the table indicate the three measured item variables with the highest factor loadings for each factor*.

The inherent reliability of the measurements was tested using Cronbach's α coefficient, and confirmatory factor analysis of the variables was performed by AMOS software. The test results are shown in Table 2. The reliability coefficients of the four variables are all greater than 0.7, indicating that the measurement scale has a high internal consistency. Compared with the standard of fitness equation model fit degree statistic (Li et al., 2018), the value-added adaptation index such as CFI is more than 0.9. Although some root mean square error of approximation (RMSEA) values exceed the 0.05 ideal threshold, they are less than the reasonable range of 0.08, indicating that the measurement scale has high validity.

**Table 2:** Reliability and validity of the measurement scale.

**Variables**	**Number of items**	**Cronbach's alpha**	**RMSEA**	**GFI**	**NFI**	**CFI**	**RFI**
Knowledge-sharing hostility	9	0.713	0.034	0.982	0.979	0.993	0.968
Knowledge manipulation	4	0.806	0.025	0.997	0.995	0.999	0.986
Coordination flexibility	4	0.890	0.018	0.997	0.998	0.999	0.993
New product development performance	5	0.710	0.061	0.988	0.963	0.976	0.926

### Descriptive statistics

Table 3 presents data on the descriptive statistics. Related controls, including region, industry and age, gender, and education, were entered first in all analyses. Explicitly, there is no significant correlation between the five control variables. There is a significant correlation between the four latent variables, such as knowledge-sharing hostility involved in the research model: knowledge-sharing hostility is significantly positively correlated with knowledge manipulation (*γ* = 0.266^**^) and significantly negatively correlated with coordination flexibility and new product development performance (*γ* = −0.135^**^, *γ* = −0.212^**^); knowledge manipulation and coordination flexibility (*γ* = −0.122^**^) and new product development performance (*γ* = −0.441^**^) have a significant negative correlation; and there is a significant positive correlation between coordination flexibility and new product development performance (*γ* = 0.302^**^).

**Table 3:** Descriptive statistics and correlation analysis of the variables.

	**Mean**	**SD**	**1**	**2**	**3**	**4**	**5**	**6**	**7**	**8**
Area	2.870	1.430								
Industry	2.880	1.400	0.017							
Age	3.100	1.019	0.086	0.088						
Gender	1.490	0.501	−0.035	0.076	0.082					
Education	3.290	1.248	−0.039	0.033	0.003	0.031				
KSH	3.568	0.484	0.050	−0.052	−0.050	−0.059	−0.030			
KM	3.583	0.778	−0.003	−0.082	0.023	0.009	0.009	0.266^**^		
CF	3.579	0.736	−0.036	0.011	−0.003	−0.054	0.021	−0.135^**^	−0.122^*^	
NPDP	3.538	0.412	0.016	0.055	−0.033	−0.011	−0.051	−0.212^**^	−0.441^**^	0.302^**^

** *indicates p < 0.01*,

* *indicates p < 0.05, two-tailed test. KSH indicates knowledge-sharing hostility, KM indicates knowledge manipulation, CF indicates coordination flexibility, and NPDP indicates new product development performance*.

### Hypothesis test

#### Main effect test

Table 4 shows the results of the regression analysis of knowledge-sharing hostility, knowledge manipulation, and new product development performance. Model 1 shows that knowledge hoarding positively affects knowledge manipulation (*β* = 0.215^***^, *p* < 0.001). Therefore, Hypothesis H_1a_ was supported. Model 2 shows that knowledge exclusion has a positive effect on knowledge manipulation (*β* = 0.152^***^, *p* < 0.001). Notably, Hypothesis H_1b_ was supported. Although the negative shared failure attitude in Model 3 reached a significant level, the overall equation failed to pass the significance test, so it is assumed that H_1c_ did not pass the verification. Model 4 illustrates that knowledge-sharing hostility significantly promotes knowledge manipulation (*β* = 0.433^***^, *p* < 0.001). Therefore, Hypothesis H_1_ was supported at the *p* < 0.001 level. Model 5 verifies that knowledge-sharing hostility significantly negatively impacts new product development performance (*β* = −0.185^**^, *p* < 0.01). Therefore, Hypothesis H_3_ was supported.

**Table 4:** Relationship between knowledge-sharing hostility and knowledge manipulation and new product development performance.

	**Model 1**	**Model 2**	**Model 3**	**Model 4**	**Model 5**	**Model 6**	**Model 7**
	**KM**	**KM**	**KM**	**KM**	**NPDP**	**NPDP**	**NPDP**
Age	0.027	0.023	0.015	0.026	−0.017	−0.009	−0.011
Gender	0.029	0.006	0.023	0.033	−0.015	−0.003	−0.008
Education level	0.007	0.005	0.009	0.010	−0.019	−0.016	−0.017
Knowledge hoarding	0.215^**^						
Knowledge exclusion		0.152^**^					
Share failure			0.131^**^				
Knowledge-sharing hostility				0.433^***^	−0.185^**^		−0.090^**^
Knowledge manipulation						−0.233^***^	−0.218^***^
R^2^	0.055	0.025	0.012	0.073	0.050	0.197	0.208
F value	6.360^***^	2.737^**^	1.314	8.501^***^	5.745^**^	26.587^***^	22.622^***^

*** *p < 0.001*,

** *p < 0.01*,

* *p < 0.05*.

#### Mediation effect test

Regarding the mediation effect test method (Wen et al., 2005), the first step is to test the impact of knowledge-sharing hostility on new product development performance through Model 5, and the results show that the independent variable significantly negatively affects the dependent variable (*β* = −0.185^**^, *p* < 0.01); the second step uses Model 4 to test the relationship between the knowledge-sharing hostility of the independent variable and the knowledge manipulation of the mediator variable and finds that the independent variable significantly positively affects the mediator variable (*β* = 0.433^***^, *p* < 0.001); the third step tests the influence of the mediator variable on the dependent variable through Model 6 and finds that knowledge manipulation significantly negatively affects new product development performance (*β* = −0.233^***^, *p* < 0.001). In the first three steps, the Model 7 analysis shows that the independent knowledge-sharing hostility and dependent variable new product development performance are still significantly related (*β* = −0.090^**^, *p* < 0.01), and there was still a significant negative correlation between intermediate variable knowledge manipulation and dependent variables (*β* = −0.218 ^***^, *p* < 0.001). The level of mediating effect is 0.433 × (−0.218) = −0.094, which is equivalent to the difference between the total effect (−0.185) and the direct effect (−0.090). Thus, it is verified that knowledge manipulation plays a partial mediating role between knowledge-sharing hostility and new product development performance, assuming H_4_ is supported.

#### Moderation effect test

Table 5 presents the results of regression analysis among knowledge manipulation, coordination flexibility, and new product development performance. Model 8 verifies that coordination flexibility positively affects new product development performance (*β* = 0.170^**^, *p* < 0.01). However, Table 3 presents the negative correlation between coordination flexibility and knowledge manipulation (*γ* = −0.122^**^, *p* < 0.01). Therefore, both provide a mathematical analysis basis for coordinating the relationship between the flexible moderation of knowledge manipulation and the performance of new product development. Based on Model 6, Model 9 further considers the “square of knowledge manipulation” and finds that it significantly negatively affects new product development performance (*β* = −0.187^***^, *p* < 0.001), indicating that knowledge manipulation has an inverted U-shaped effect on new product development performance. Therefore, Hypothesis H_2_ was supported.

**Table 5:** Relationship test between knowledge manipulation, coordination flexibility and new product development performance.

	**Model 8**	**Model 9**	**Model 10**	**Model 11**	**Model 12**	**Model 13**	**Model14**
	**NPDP**	**NPDP**	**NPDP**	**NPDP**	**NPDP**	**NPDP**	**NPDP**
Age	−0.013	−0.020	−0.014	0.009	0.004	−0.037	−0.025
Gender	0.008	−0.014	0.000	−0.053	−0.054	0.021	0.007
Education	−0.019	−0.011	−0.015	−0.031	−0.029	−0.140	−0.009
Knowledge manipulation		−0.305^***^	−0.291^**^	−0.273^***^	−0.284^***^	−0.139^***^	−0.314^***^
Square of knowledge manipulation		−0.187^***^	−0.164^***^		−0.036		−0.298^***^
Coordination flexibility	0.170^**^		0.206^***^				
Knowledge manipulation × coordination flexibility			−0.020^***^				
Knowledge manipulation square × coordination flexibility			−0.122^***^				
R^2^	0.095	0.348	0.441	0.331	0.337	0.083	0.478
▵ R^2^			0.093				
F value	11.423^***^	46.125^***^	42.318^***^	24.171^***^	19.712^***^	5.264^***^	42.438^***^

*** *Indicates p < 0.001*,

** *indicates p < 0.01*,

* *indicates p < 0.05; models 8, 9, and 10 are 438 samples, and models 11 and 12 are 200 samples with low coordination flexibility. Models 13 and 14 are 238 sample sets under high coordination flexibility*.

Model 10 is used to test the moderation effect of coordination flexibility between knowledge manipulation and new product development performance. We find that knowledge manipulation negatively affects new product development performance (*β* = −0.291^***^, *p* < 0.001) and coordination flexibility positively affects new product development performance (*β* = 0.206^***^, *p* < 0.001). Moreover, the regression coefficient of knowledge manipulation and coordination of flexible interaction terms for new product development performance is *β* = −0.020^***^, and the regression coefficient between knowledge manipulation square and coordinated flexibility to new product development performance is *β* = −0.122^***^, Δ*R*^2^ = 0.093. Therefore, coordination flexibility plays a moderating role in the relationship between knowledge manipulation and new product development performance. Therefore, Hypothesis H_5_ was supported.

**Figure 2:**
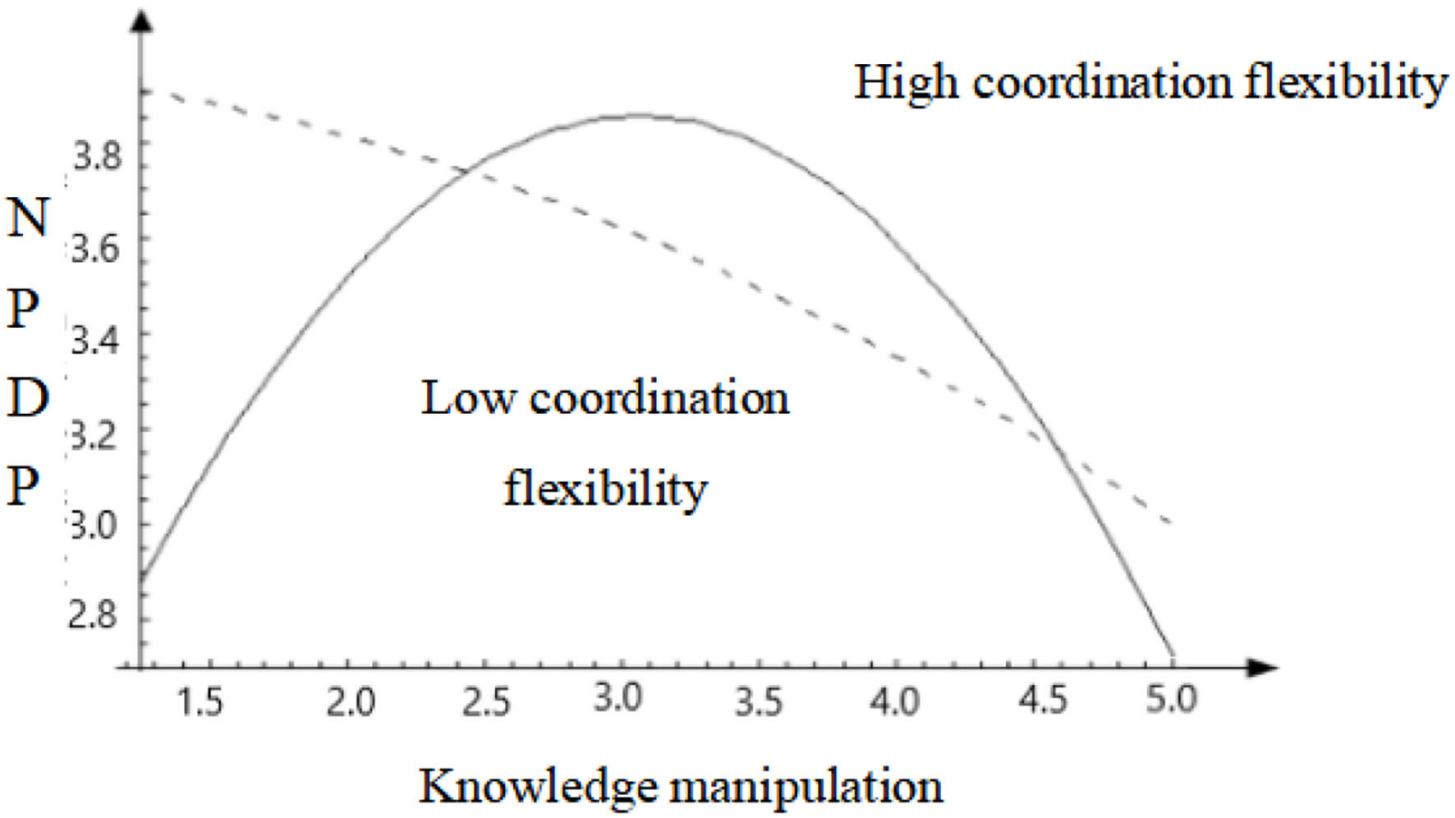
Moderating effect of coordination flexibility.

To further illustrate the moderating role of flexibility coordination, 438 samples were divided into low or high coordination flexibility groups according to the mean value of coordination flexibility of 3.579. Model 11 shows that knowledge manipulation under low coordination significantly negatively affects the performance of new product development (*β* = −0.273^***^, *p* < 0.001). Based on this, Model 12 introduces the “square of knowledge manipulation” and finds that knowledge manipulation still has a significant negative impact on new product development performance (*β* = −0.284^***^, *p* < 0.001), but the “square of knowledge manipulation” does not pass the significance test, indicating that knowledge manipulation under low coordination flexibility is only monotonous and negatively affects new product development performance. Model 13 is the regression result of knowledge manipulation to predict new product development performance under high coordination flexibility. At this time, the coefficient of knowledge manipulation is (*β* = −0.139^***^, *p* < 0.001), but the coefficient of judgment is small (*R*^2^ = 0.083). This shows that knowledge manipulation under high coordination flexibility can still predict the change in new product development performance to a small extent. Model 14 continues to introduce the “square of knowledge manipulation” and finds that the equation as a whole passes the significance test. The coefficients of knowledge manipulation and its squared term are −0.314^***^ and −0.298^***^, respectively, indicating an inverted U-shaped relationship between knowledge manipulation and new product development performance under high coordination, and Hypothesis H_5_ is supported. The pattern is shown in Figure 2.

#### Moderated mediation effect test

According to Hayes's suggestion (Hayes, 2013), we found that knowledge manipulation mediates the relationship of knowledge-sharing hostility on the performance of new product development. Second, coordination flexibility moderates the relationship between knowledge manipulation and new product development performance (*β* = −0.122^***^). Notably, Hypothesis H_5_ was supported. Third, we test the moderated mediation effects using the bootstrap method and select the 14th model. The specific results are shown in Table 6.

**Table 6:** Mediating effect of knowledge manipulation on different levels of coordination flexibility.

**Independent variables**	**Moderator**	**Indirect effect**	**Standard error**	**95% confidence interval**	
Knowledge-sharing hostility	Low	−0.115^*^	0.025	−0.173	−0.070
	Medium	−0.083^*^	0.023	−0.134	−0.046
	High	−0.050	0.026	−0.113	0.012
	Index of moderated mediation	0.044^*^	0.016	0.015	0.079

*** *p < 0.001*,

** *p < 0.01*,

* *p < 0.05; the low, medium, and high values of coordination flexibility refer to the mean minus one standard deviation, the mean, the mean plus one standard deviation*.

Table 6 shows that the moderation index of coordination flexibility on knowledge manipulation between knowledge-sharing hostility and new product development performance is significant (regulation index is 0.044, *p* < 0.05), and the 95% confidence interval does not contain zero. At low levels of coordination flexibility, the 95% confidence interval does not contain zero (−0.173, −0.070), indicating that the mediating effect of knowledge manipulation is significant. Similarly, the indirect effects are also significant in the condition of coordination flexibility at the middle or high level. Therefore, Hypothesis H_6_ was supported.

## Discussion

Knowledge is critical for new product development performance, and increasing importance highlights the motivational dilemma of knowledge sharing. Based on the theory of knowledge-sharing hostility and knowledge manipulation, this paper constructs a moderated mediation model and tests the relevant hypotheses with 438 survey data. We found that knowledge hoarding and knowledge exclusion positively affect knowledge manipulation behavior, but there is no correlation between negative sharing failure attitude and knowledge manipulation. Moreover, the hostility of knowledge sharing, which consists of knowledge hoarding, knowledge exclusion, and negative sharing failure attitudes, affects new product development performance, and knowledge manipulation mediates the relationship between the hostility of knowledge sharing and new product development performance. In addition, coordination flexibility moderates the relationship between knowledge manipulation and new product development performance. Specifically, when coordination flexibility is low, knowledge manipulation negatively affects new product development performance; however, when coordination flexibility is high, there is an inverted U-shaped curve relationship between knowledge manipulation and new product development performance. Furthermore, we found a mediating effect of knowledge-sharing hostility on new product development performance, although knowledge manipulation is moderated by coordination flexibility. In summary, it is necessary to attach great importance to improve product innovation performance through high-level coordination and flexible knowledge management.

### Theoretical contributions

The academic significance of this study can be elaborated as follows. First, our research has enriched the theory of knowledge-sharing hostility. Explicitly, prior studies have focused on the antecedent variables and the relationship with knowledge sharing. This paper verifies that knowledge-sharing hostility mainly includes three dimensions: knowledge hoarding, knowledge exclusion, and negative sharing failure attitudes. We explore the mechanism of knowledge-sharing hostility on the performance of new product development. In other words, the hostility of knowledge sharing affects new product development performance, and knowledge manipulation mediates the relationship between the hostility of knowledge sharing and new product development performance.

Second, this research enriches knowledge management theory and provides a new perspective for the improvement in innovation performance. Since innovation is the process of knowledge gathering and analysis, it can help to promote the speed of innovation and the quality of innovation by obtaining a large amount of knowledge stock and knowledge flow (Ohlsson, 2011). However, knowledge workers usually choose knowledge manipulation due to many factors, such as personality, incentive mechanisms, goal orientation, and exchange awareness. Coordination flexibility can moderate the mediating role of knowledge manipulation between knowledge-sharing hostility and new product development performance. Specifically, this paper verifies that there is an inverted U-shaped relationship between knowledge manipulation and new product development performance under high coordination flexibility, which essentially explains the positive significance of low-level knowledge manipulation and the negative impact of high-level knowledge manipulation.

### Practical implications

The practical implications of this study are as follows. First, the development of new products in different industries requires that employees cooperate with each other. However, the dilemma of knowledge sharing is widespread due to the incompatibility of goals, the differences in knowledge ownership perception, and the individual characteristics of members. In practice, enterprises pay attention to material awards, and neglecting material rewards may hinder individual knowledge-sharing behavior (Liu et al., 2017). This study provides new insights by identifying the mechanism of knowledge-sharing hostility and knowledge manipulation on the development performance of new products. It can be argued that the transaction-based incentive mechanism induces negative individual knowledge management behaviors such as knowledge manipulation and thus negatively affects innovation performance. Accordingly, it does not advocate excessive preference for the material reward at the individual level.

Second, this paper reveals the impact of knowledge manipulation on the performance of new product development. Prior research compared the motivation and dilemma of knowledge sharing (Kimmerle and Wodzicki, 2011). Combined with the research conclusions of this paper, it can provide a more pragmatic choice by considering the discussion of new product innovation performance based on the knowledge-sharing hostility, knowledge manipulation, and other negative individual knowledge management. In practice, knowledge-sharing hostility is relatively easy to discern and correct; however, the positive significance of suitable knowledge manipulation has been proven to be of positive significance, but it is easy to ignore (Erdem and Mustafa, 2009; Rhee and Choi, 2017). Therefore, it is necessary to control the knowledge manipulation of knowledge workers at the middle or low level and to solve the dilemma of knowledge sharing with the help of a medium or high level of coordination and flexibility.

### Limitations and future research directions

Despite its theoretical and practical implications, our study has the following limitations. First, research on the relationship among knowledge sharing, knowledge hiding, and innovation performance has received widespread attention. Although this paper attempts to innovate from the perspective of knowledge-sharing hostility and knowledge manipulation, the three individual knowledge management behaviors of knowledge sharing, knowledge hiding, and knowledge manipulation are not included in the overall research framework, so further research can still be carried out. Second, our study could still be deviated by the attributions of the respondents, since necessary modifications were made to the questionnaire. Finally, future research can add more related antecedents or result variables based on this paper to conduct more in-depth knowledge management behavior research.

## Data availability statement

The raw data supporting the conclusions of this article will be made available by the authors, without undue reservation.

## Author contributions

RC developed the theoretical framework and worked on literature review, data collection, and manuscript writing. YM worked on manuscript writing and revising. Both authors contributed to the article and approved the submitted version.

## Funding

This study was supported by the major decision-making consultation project of Shanxi Provincial Government (2020ZB12005).

## Conflict of interest

The authors declare that the research was conducted in the absence of any commercial or financial relationships that could be construed as a potential conflict of interest.

## Publisher's note

All claims expressed in this article are solely those of the authors and do not necessarily represent those of their affiliated organizations, or those of the publisher, the editors and the reviewers. Any product that may be evaluated in this article, or claim that may be made by its manufacturer, is not guaranteed or endorsed by the publisher.
